# A New Kind of Fuzzy *n*-ary Hypergroups in the Framework of Soft Set Theory

**DOI:** 10.1155/2014/957391

**Published:** 2014-07-14

**Authors:** Hongjie Li, Yunqiang Yin

**Affiliations:** ^1^Mathematics Department of Zhoukou Normal University, Zhoukou 466001, China; ^2^School of Sciences, East China Institute of Technology, Nanchang 330013, China

## Abstract

Maji et al. introduced the concept of fuzzy soft sets as a generalization of the standard soft sets and presented an application of fuzzy soft sets in a decision making problem. The aim of this paper is to apply the concept of fuzzy soft sets to *n*-ary hypergroup theory. The concepts of (∈_*γ*_, ∈_*γ*_∨*q*_*δ*_)-fuzzy soft (invertible) *n*-ary subhypergroups over a commutative *n*-ary hypergroup are introduced and some related properties and characterizations are obtained. The homomorphism properties of (∈_*γ*_, ∈_*γ*_∨*q*_*δ*_)-fuzzy soft (invertible) *n*-ary subhypergroups are also derived.

## 1. Introduction

To solve complicated problems in economics, engineering, and environment, we cannot successfully use classical methods because of various uncertainties typical for those problems. There are three theories: theory of probability, theory of fuzzy sets, and the interval mathematics, which we can consider as mathematical tools for dealing with uncertainties. However, all of these have their advantages as well as inherent limitations in dealing with uncertainties. One major problem shared by those theories is their incompatibility with the parameterization tools. To overcome these difficulties, Molodtsov [1] introduced the concept of soft set as a new mathematical tool for dealing with uncertainties that is free from the difficulties that have troubled the usual theoretical approaches. Molodtsov pointed out several directions for the applications of soft sets. This theory has proven useful in many different fields such as decision making [2–6], data analysis [7, 8], and forecasting [9].

Up to the present, research on soft sets has been very active and many important results have been achieved in the theoretical aspect. Maji et al. [10] introduced several algebraic operations in soft set theory and published a detailed theoretical study on soft sets. Ali et al. [11] further presented and investigated some new algebraic operations for soft sets. Qin and Hong [12] further investigated the algebraic structure of soft sets and established the soft quotient algebra. Maji et al. [13] and Majumdar and Samanta [14] extended (classical) soft sets to fuzzy soft sets, respectively. Maji et al. [15] extended (classical) soft sets to intuitionistic fuzzy soft sets, which were further discussed by Maji et al. [16] and Jiang et al. [17]. Aktaş and Çağman [18] compared soft sets to the related concepts of fuzzy sets and rough sets. They also defined the notion of soft groups and derived some related properties. Aygünoğlu and Aygün [19] discussed the applications of fuzzy soft sets to group theory and investigated (normal) fuzzy soft groups. Feng et al. [20] investigated soft semirings by using the soft set theory. Jun [21] introduced and investigated the notion of soft BCK/BCI-algebras. Jun and Park [22] and Jun et al. [23] discussed the applications of soft sets in ideal theory of BCK/BCI-algebras and in *d*-algebras, respectively. Acar et al. [24] introduced and studied soft rings. Zhan and Jun [25] characterized the (implicative, positive implicative, and fantastic) filteristic soft *BL*-algebras based on ∈-soft sets and *q*-soft sets.

On the other hand, the fuzzy sets and hyperstructures introduced by Zadeh and Marty, respectively, are now extensively applied to many disciplines. The study of fuzzy hyperstructures is an interesting research topic of fuzzy sets. There is a considerable amount of work on the connections between fuzzy sets and hyperstructures. The reader is referred to [26–38]. As a generalization of the concepts of “belongingness (∈)” and “quasi-coincidence (*q*)” of a fuzzy point with a fuzzy set introduced by Pu and Liu [39], Yin and Zhan [40] introduced the concepts of “*γ*-belongingness (∈_*γ*_)” and “*δ*-quasi-coincidence (*q*_*δ*_)” of a fuzzy point with a fuzzy set. Using these new concepts, Yin and Zhan [40] introduced the concepts of (*α*, *β*)-fuzzy (implicative, positive implicative, and fantastic) filters and $\left(\bar{\beta },\bar{\alpha }\right)$-fuzzy (implicative, positive implicative, and fantastic) filters of *BL*-algebras, where *α*, *β* ∈ {∈_*γ*_, *q*_*δ*_, ∈_*γ*_∧*q*_*δ*_, ∈_*γ*_∨*q*_*δ*_}, $\bar{\alpha },\bar{\beta }\in \left\{\bar{\in_{\gamma }},\bar{q_{\delta }},\bar{\in_{\gamma }}\wedge \bar{q_{\delta }},\bar{\in_{\gamma }}\vee \bar{q_{\delta }}\right\}$, *α* ≠ ∈_*γ*_∧*q*_*δ*_, and $\bar{\beta }\neq \bar{\in_{\gamma }}\wedge \bar{q_{\delta }}$, and some related properties were investigated. Special attention is paid to $\left(\bar{\in_{\gamma }},\bar{\in_{\gamma }}\vee \bar{q_{\delta }}\right)$-fuzzy (implicative, positive implicative, and fantastic) filters and $\left(\bar{\in_{\gamma }},\bar{\in_{\gamma }}\vee \bar{q_{\delta }}\right)$-fuzzy (implicative, positive implicative, and fantastic) filters which are generalizations of (∈, ∈∨ *q*)-fuzzy (implicative, positive implicative, and fantastic) filters and $\left(\bar{\in },\bar{\in }\vee \bar{q}\right)$-fuzzy (implicative, positive implicative, and fantastic) filters studied by Ma et al. [41, 42]. This idea is continued and studied by Ma et al. [43], Yin and Zhan [44, 45], Yin et al. [46], and so on. The purpose of this paper is to deal with the algebraic structure of *n*-ary hypergroups by applying fuzzy soft set theory. We introduce the concepts of (∈_*γ*_, ∈_*γ*_∨*q*_*δ*_)-fuzzy soft (invertible) *n*-ary subhypergroups over a commutative *n*-ary hypergroup and focus on investigating their characterization and algebraic properties. We also discuss the homomorphism properties of (∈_*γ*_, ∈_*γ*_∨*q*_*δ*_)-fuzzy soft (invertible) *n*-ary subhypergroups.

## 2. Preliminaries

In this section, we recall some basic notions and results about *n*-ary hypergroups, fuzzy sets, and fuzzy soft sets (see [13, 28, 30, 47]).

### 2.1. *n*-Ary Hypergroups

We will be concerned primarily with a basic nonempty set *H*. Denote by *H* ^*n*^ the cartesian product *H* × ⋯×*H*, where *H* appears *n* times. An element of *H* ^*n*^ will be denoted by (*x*_1_,…, *x*_*n*_), where *x*_*i*_ ∈ *H* for all 1 ≤ *i* ≤ *n*. In general, a mapping *f* : *H* ^*n*^ → P*(*H*) is called an *n-ary hyperoperation* and *n* is called the* arity* of the hyperoperation *f*. Let *f* be an *n*-ary hyperoperation on *H* and *A*_1_,…, *A*_*n*_ subsets in *H*. Define

(1) $$\begin{array}{c} f\left(A_{1},\ldots ,A_{n}\right)=\cup \left\{f\left(x_{1},\ldots ,x_{n}\right)\;|\;x_{i}\in A_{i},\;\;1\leq i\leq n\right\}. \end{array}$$

In the sequel, we will denote the sequence *x*_*i*_, *x*_*i*+1_,…, *x*_*j*_ by *x*_*i*_^*j*^. For *j* < *i*, *x*_*i*_^*j*^ = *∅*. Thus,

(2) $$\begin{array}{c} f\left(x_{1},\ldots ,x_{i},y_{i+1},\ldots ,y_{j},z_{j+1},\ldots ,z_{n}\right) \end{array}$$

will be written as *f*(*x*_1_^*i*^, *y*_*i*+1_^*j*^, *z*_*j*+1_^*n*^). Also *f*(*a*_1_^*i*^, *x*∗) means $f\left(a_{1}^{i},\underset{n-i}{\underset{︸}{x,\ldots ,x}}\right)$ for *a*_1_^*i*^, *x* ∈ *H* and 1 ≤ *i* ≤ *n*.

*H* with an *n*-ary hyperoperation *f* : *H* ^*n*^ → P*(*H*) is called an *n-ary hypergroupoid* and will be denoted by (*H*, *f*). An *n*-ary hypergroupoid (*H*, *f*) is said to be an *n-ary semihypergroup* if the following associative axiom holds:

(3) $$\begin{array}{c} f\left(x_{1}^{i-1},f\left(x_{i}^{n+i-1}\right),x_{n+i}^{2n-1}\right)=f\left(x_{1}^{j-1},f\left(x_{j}^{n+j-1}\right),x_{n+j}^{2n-1}\right) \end{array}$$

for all *i*, *j* ∈ {1,…, *n*} and *x*_1_^2*n*−1^ ∈ *H*. An *n*-ary semihypergroup is said to be an *n-ary hypergroup* if, for all *y*, *y*_1_^*n*^ ∈ *H* and fixed *i* ∈ {1,…, *n*}, there exists *x* ∈ *H* such that *y* ∈ *f*(*y*_1_^*i*−1^, *x*, *y*_*i*+1_^*n*^). An *n*-ary hypergroup is said to be* commutative* if, for all *x*_1_^*n*^ ∈ *H* and any permutation *ρ* of {1,…, *n*}, we have *f*(*x*_1_^*n*^) = *f*(*x*_*ρ*_1__,…, *x*_*ρ*_*n*__). An element *e* of *H* is called an* identity* of *H* if, for any *x* ∈ *H* and *i* ∈ {1,…, *n*}, we have $x\in f\left(\underset{i-1}{\underset{︸}{e,\ldots ,e}},x,\underset{n-i}{\underset{︸}{e,\ldots ,e}}\right)$.

**Definition 1 .**

Let (*H*, *f*) be an *n*-ary hypergroup and *K* a nonempty subset of *H*. One says that *K* is an *n*-*ary subhypergroup* of *H* if the following conditions hold:

- *K*  is closed under the *n*-ary hyperoperation *f*;
- for all *y*, *y*_1_^*n*^ ∈ *K* and fixed *i* ∈ {1,…, *n*}, there exists *x* ∈ *K* such that *y* ∈ *f*(*y*_1_^*i*−1^, *x*, *y*_*i*+1_^*n*^).

In what follows, (*H*, *f*) denotes a commutative *n*-ary hypergroup with an identity *e* unless otherwise stated.

An *n*-ary subhypergroup *K* of *H* is said to be* invertible* if, for any *x*, *y* ∈ *H*, $x\in f\left(y,\underset{n-1}{\underset{︸}{K,\ldots ,K}}\right)$ implies $y\in f\left(x,\underset{n-1}{\underset{︸}{K,\ldots ,K}}\right)$.

### 2.2. Fuzzy Sets

Let *X* be a nonempty set. A fuzzy subset *μ* of *X* is defined as a mapping from *X* into [0,1], where [0,1] is the usual interval of real numbers. We denote by *F*(*X*) the set of all fuzzy subsets of *X*. For *μ*, *ν* ∈ *F*(*X*), by *μ*⊆*ν*, we mean *μ*(*x*) ≤ *ν*(*x*) for all *x* ∈ *X*. And the* union* and* intersection* of *μ* and *ν*, denoted by *μ* ∪ *ν* and *μ*∩*ν*, are defined as the fuzzy subsets of *X* by (*μ* ∪ *ν*)(*x*) = max⁡{*μ*(*x*), *ν*(*x*)} and (*μ*∩*ν*)(*x*) = min⁡{*μ*(*x*), *ν*(*x*)} for all *x* ∈ *X*.

For *x*_*i*_, *x*_*i*+1_,…, *x*_*j*_ ∈ *X* and *μ* ∈ *F*(*X*), the sequence *μ*(*x*_*i*_), *μ*(*x*_*i*+1_),…, *μ*(*x*_*j*_) is denoted by *μ*_*x*_*i*__^*x*_*j*_^, and *μ*_*x*_*i*__^*x*_*j*_^ = 0 if *j* < *i*. Moreover, for *μ*_*i*_, *μ*_*i*+1_,…, *μ*_*j*_ ∈ *F*(*X*), the sequence *μ*_*i*_, *μ*_*i*+1_,…, *μ*_*j*_ is denoted by *μ*_*i*_^*j*^, and *μ*_*i*_^*j*^ denotes the zero fuzzy set if *j* < *i*.

For any *Y*⊆*X* and *r* ∈ (0,1], define a fuzzy subset *r*_*Y*_ of *X* by

(4) rY(x)={r  if  x∈Y,0otherwise,

for all *x* ∈ *X*. In particular, when *r* = 1, *r*_*Y*_ is said to be the* characteristic function* of *Y*, denoted by *χ*_*Y*_, and when  *X* = {*x*}, *r*_*X*_ is said to be a* fuzzy point* with support *x* and value *r* and is denoted by *r*_*x*_.

In what follows, let *γ*, *δ* ∈ [0,1] be such that *γ* < *δ*. For a fuzzy point *r*_*x*_ and a fuzzy subset *μ* of *X*, we say that

1. *r*_*x*_∈_*γ*_ *μ* if *μ*(*x*) ≥ *r* > *γ*;
2. *r*_*x*_ *q*_*δ*_ *μ* if *μ*(*x*) + *r* > 2*δ*;
3. *r*_*x*_ ∈_*γ*_ ∨*q*_*δ*_ *μ* if *r*_*x*_ ∈_*γ*_ *μ* or *r*_*x*_ *q*_*δ*_ *μ* [40].

Let us now introduce a new relation on *F*(*X*), denoted by “⊆∨*q*_(*γ*,*δ*)_,” as follows.

For any *μ*, *ν* ∈ *F*(*X*), by *μ*⊆∨*q*_(*γ*,*δ*)_ *ν*, we mean that *r*_*x*_∈_*γ*_ *μ* implies *r*_*x*_∈_*γ*_∨*q*_*δ*_ *ν* for all *x* ∈ *X* and *r* ∈ (*γ*, 1]. Moreover, *μ* and *ν* are said to be (*γ*, *δ*)-equal, denoted by *μ*=_(*γ*,*δ*)_ *ν*, if *μ*⊆∨*q*_(*γ*,*δ*)_ *ν* and *ν*⊆∨*q*_(*γ*,*δ*)_ *μ*.

In the sequel, unless otherwise stated, $\bar{\alpha }$ means that *α* does not hold, where *α* ∈ {∈_*γ*_, *q*_*δ*_, ∈_*γ*_∨*q*_*δ*_, ⊆∨*q*_(*γ*,*δ*)_}.

**Lemma 2 (see [40]).**

Let *μ*, *ν* ∈ *F*(*X*). Then *μ*⊆∨*q*_(*γ*,*δ*)_ *ν* if and only if max⁡{*ν*(*x*), *γ*} ≥ min⁡{*μ*(*x*), *δ*} for all *x* ∈ *X*.

**Lemma 3 (see [40]).**

Let *μ*, *ν*, *ω* ∈ *F*(*X*). If *μ*⊆∨*q*_(*γ*,*δ*)_ *ν* and  *ν*⊆∨*q*_(*γ*,*δ*)_ *ω*, then *μ*⊆∨*q*_(*γ*,*δ*)_ *ω*.

**Proof**

It is straightforward by Lemma 2

Lemmas 2 and 3 give that “=_(*γ*,*δ*)_” is an equivalence relation on *F*(*X*). It is also worth noticing that *μ*=_(*γ*,*δ*)_ *ν* if and only if max⁡{min⁡{*μ*(*x*), *δ*}, *γ*} = max⁡{min⁡{*ν*(*x*), *δ*}, *γ*} for all *x* ∈ *X* by Lemma 2

### 2.3. Fuzzy Soft Sets

Let *U* be an initial universe set and *E* the set of all possible parameters under consideration with respect to *U*. As a generalization of soft set introduced by Molodtsov [1], Maji et al. [13] defined fuzzy soft set in the following way.

**Definition 4 .**

A pair 〈*F*, *A*〉 is called a* fuzzy soft set* over *U*, where *A*⊆*E* and *F* is a mapping given by *F* : *A* → *F*(*U*).

In general, for every *ε* ∈ *A*, *F*(*ε*) is a fuzzy set of *U* and it is called* fuzzy value set* of parameter *ε*. The set of all fuzzy soft sets over *U* with parameters from *E* is called* fuzzy soft classes*, and it is denoted by *FS*(*U*, *E*). A fuzzy soft set 〈*F*, *A*〉 in *FS*(*U*, *E*) is said to* have sup-property* if, for any *V* ∈ P*(*U*) and *ε* ∈ *A*, there exists *x*_0_ ∈ *V* such that *F*(*ε*)(*x*_0_) = sup⁡_*x*∈*V*_⁡*F*(*ε*)(*x*).

Let *A*⊆*E* and *μ* ∈ *F*(*U*). Define $\langle \tilde{\mu },A\rangle \;$to be a fuzzy soft set by $\tilde{\mu }(\varepsilon )=\mu$ for all *ε* ∈ *A*. For any fuzzy point *r*_*x*_ in *U*, define $\left(\tilde{r_{x}},A\right)$to be a fuzzy soft set by $\tilde{r_{x}}(\varepsilon )=r_{x}$ for all *ε* ∈ *A*.

**Definition 5 (see [6]).**

Let 〈*F*, *A*〉 and 〈*G*, *B*〉 be two fuzzy soft sets over  *U*. One says that 〈*F*, *A*〉 is a* fuzzy soft subset* of 〈*G*, *B*〉 and writes 〈*F*, *A*〉⋐〈*G*, *B*〉 if

- *A*⊆*B*;
- for any *ε* ∈ *A*, *F*(*ε*)⊆*G*(*ε*).

〈*F*, *A*〉 and 〈*G*, *B*〉 are said to be* fuzzy soft equal* and write 〈*F*, *A*〉 = 〈*G*, *B*〉 if 〈*F*, *A*〉⋐〈*G*, *B*〉 and 〈*G*, *B*〉⋐〈*F*, *A*〉.

Let us now introduce some new concepts on fuzzy soft sets analogous to the concepts introduced by Ali et al. [11].

**Definition 6 .**

The* extended intersection* of two fuzzy soft sets 〈*F*, *A*〉 and 〈*G*, *B*〉 over *U* is a fuzzy soft set denoted by 〈*H*, *C*〉, where *C* = *A* ∪ *B* and

(5) $$\begin{array}{c} H\left(\varepsilon \right)=\{\begin{array}{cc} F\left(\varepsilon \right) & \text{if}\;\varepsilon \in A-B,\\ G\left(\varepsilon \right) & \text{if}\;\varepsilon \in B-A,\\ F\left(\varepsilon \right)\cap G\left(\varepsilon \right) & \text{if}\;\varepsilon \in A\cap B, \end{array} \end{array}$$

for all *ε* ∈ *C*. This is denoted by $\langle H,C\rangle =\langle F,A\rangle \tilde{\cap }\langle G,B\rangle$.

**Definition 7 .**

Let 〈*F*, *A*〉 and 〈*G*, *B*〉 be two fuzzy soft sets over *U* such that *A*∩*B* ≠ *∅*. The* restricted intersection* of 〈*F*, *A*〉 and 〈*G*, *B*〉 is defined to be the fuzzy soft set 〈*H*, *C*〉, where *C* = *A*∩*B* and *H*(*ε*) = *F*(*ε*)∩*G*(*ε*) for all *ε* ∈ *C*. This is denoted by 〈*H*, *C*〉 = 〈*F*, *A*〉⋒〈*G*, *B*〉.

**Definition 8 .**

Let *V*⊆*U* and *r* ∈ (*γ*, 1]. A fuzzy soft set 〈*F*, *A*〉 over *V* is said to be a* relative whole *(*γ*, *δ*)-*fuzzy soft set* (with respect to universe set *V*, parameter set *A*, and value *r*), denoted by Σ(*V*, *A*, *r*), if *F*(*ε*) = *r*_*V*_ for all *ε* ∈ *A*.

**Definition 9 .**

Let 〈*F*, *A*〉 and 〈*G*, *B*〉 be two fuzzy soft sets over *U*. One says that 〈*F*, *A*〉 is (*γ*, *δ*)-*fuzzy soft subset* of 〈*G*, *B*〉and writes 〈*F*, *A*〉⋐_(*γ*,*δ*)_〈*G*, *B*〉 if

- *A*⊆*B*;
- for any *ε* ∈ *A*, *F*(*ε*)⊆∨*q*_(*γ*,*δ*)_ *G*(*ε*).

〈*F*, *A*〉 and 〈*G*, *B*〉 are said to be (*γ*, *δ*)-*fuzzy soft equal* and write 〈*F*, *A*〉≍_(*γ*,*δ*)_〈*G*, *B*〉 if 〈*F*, *A*〉⋐_(*γ*,*δ*)_〈*G*, *B*〉 and 〈*G*, *B*〉⋐_(*γ*,*δ*)_〈*F*, *A*〉.

Clearly, 〈*F*, *A*〉⋐〈*G*, *B*〉 implies 〈*F*, *A*〉⊆∨*q*_(*γ*,*δ*)_〈*G*, *B*〉 by Lemma 2 and Definition 9.

**Lemma 10 .**

Let 〈*F*, *A*〉, 〈*G*, *B*〉, and 〈*H*, *C*〉 be fuzzy soft sets over *U*. If 〈*F*, *A*〉⋐_(*γ*,*δ*)_〈*G*, *B*〉 and 〈*G*, *B*〉⋐_(*γ*,*δ*)_〈*H*, *C*〉, then 〈*F*, *A*〉⋐_(*γ*,*δ*)_〈*F*, *C*〉.

**Proof**

It is straightforward by Lemma 3 and Definition 9.

Lemmas 2 and 10 and Definition 9 give that “≍_(*γ*,*δ*)_” is an equivalence relation on *FS*(*U*, *E*).

## 3. A New Operation on Fuzzy Soft Sets over an *n*-Ary Hypergroupoid

We first introduce a fuzzy *n*-ary hyperoperation on an *n*-ary hypergroupoid as follows.

**Definition 11 .**

Let (*H*, *f*) be an *n*-ary hypergroupoid and *μ*_1_,…, *μ*_*n*_ ∈ *F*(*H*). Define a fuzzy *n*-ary hyperoperation $F:\underset{n}{\underset{︸}{F\left(H\right)\times \cdots \times F(H)}}\to F(H)$ by

(6) $$\begin{array}{c} F\left(\mu_{1},\ldots ,\mu_{n}\right)\left(x\right)=\underset{x\in f\left(y_{1}^{n}\right)}{\sup}\min\left\{\mu_{1}\left(y_{1}\right),\ldots ,\mu_{n}\left(y_{n}\right)\right\} \end{array}$$

for all *x* ∈ *H*. In particular, for any *x*_1_^*n*^ ∈ *H* and *i* ∈ {1,…, *n*}, define

(7) F(μ1,…,μi,xi+1n)(x) =sup⁡x∈f(y1i,xi+1n)min⁡{μ1(y1),…,μi(yi)}

for all *x* ∈ *H*.

Clearly, for any *x*_1_^*n*^ ∈ *H* and 1 ≤ *i* ≤ *n*, *F*(*μ*_1_,…, *μ*_*i*_, *x*_*i*+1_^*n*^) = *F*(*μ*_1_,…, *μ*_*i*_, 1_*x*_*i*+1__,…, 1_*x*_*n*__). Note that, for any *r*_1_^*n*^ ∈ (0,1] and *x*_1_^*n*^ ∈ *H*, by Definition 11, *F*(*r*_1_*x*_1___, *r*_2_*x*_2___,…, *r*_*n*_*x*_*n*___) = min⁡{*r*_1_^*n*^}_*f*(*x*_1_^*n*^)_.

**Definition 12 .**

Let (*H*, *f*) be an *n*-ary hypergroupoid and 〈*F*_*i*_, *A*_*i*_〉 ∈ *FS*(*H*, *E*), where *i* ∈ *I* and *I* is an index set, such that ⋂_*i*∈*I*_ *A*_*i*_ ≠ *∅*. One defines a fuzzy soft set over *H*, denoted by 〈*F*(*F*_1_,…, *F*_*n*_), *C*〉, where *C* = ⋂_*i*∈*I*_ *A*_*i*_ and

(8) $$\begin{array}{c} F\left(F_{1},\ldots ,F_{n}\right)\left(\varepsilon \right)=F\left(F_{1}\left(\varepsilon \right),\ldots ,F_{n}\left(\varepsilon \right)\right), \end{array}$$

for all *ε* ∈ *C*. This is denoted by 〈*F*(*F*_1_,…, *F*_*n*_), *C*〉 = *F*(〈*F*_1_, *A*_1_〉,…, 〈*F*_*n*_, *A*_*n*_〉).

In the following, we will denote the sequence 〈*F*_*i*_, *A*_*i*_〉, 〈*F*_*i*+1_, *A*_*i*+1_〉,…, 〈*F*_*j*_, *A*_*j*_〉 by 〈*F*,*A*〉_*i*_^*j*^. For *j* < *i*, 〈*F*,*A*〉_*i*_^*j*^ is the empty set. The following elementary facts follow easily from the definition.

**Lemma 13 .**

Let (*H*, *f*) be an *n*-ary hypergroupoid and 〈*F*,*A*〉_1_^2*n*−1^, 〈*G*,*B*〉_1_^*n*^ ∈ *FS*(*H*, *E*). Then

(1) if (*H*, *f*) is an *n*-ary semihypergroup and ⋂_*i*=1_^2*n*^ *A*_*i*_ ≠ *∅*, then
  (9) F(〈F,A〉1i−1,F(〈F,A〉in+i−1),〈F,A〉n+i2n−1) =F(〈F,A〉1j−1,F(〈F,A〉jn+j−1),〈F,A〉n+j2n−1)
  for all 1 ≤ *i*, *j* ≤ *n*;
(2) if (*H*, *f*) is commutative and ⋂_*i*=1_^*n*^ *A*_*i*_ ≠ *∅*, then the value of *F*(〈*F*, *A*〉_1_^*n*^) does not depend on the permutation of 〈*F*_1_, *A*_1_〉,…, 〈*F*_*n*_, *A*_*n*_〉;
(3) if 〈*F*_*i*_, *A*_*i*_〉⋐_(*γ*,*δ*)_〈*G*_*i*_, *B*_*i*_〉 for all 1 ≤ *i* ≤ *n* and ⋂_*i*=1_^*n*^ *A*_*i*_ ≠ *∅*, then *F*(〈*F*, *A*〉_1_^*n*^)⋐_(*γ*,*δ*)_ *F*(〈*G*, *B*〉_1_^*n*^);
(4) if 〈*F*_1_, *A*_1_〉⋐_(*γ*,*δ*)_〈*G*_1_, *A*_1_〉 and  〈*F*_2_, *A*_2_〉⋐_(*γ*,*δ*)_〈*G*_2_, *A*_2_〉, then $\langle F_{1},A_{1}\rangle \tilde{\cap }\langle F_{2},A_{2}\rangle {⋐}_{\left(\gamma ,\delta \right)}\langle G_{1},A_{1}\rangle \tilde{\cap }\langle G_{2},A_{2}\rangle$ and 〈*F*_1_, *A*_1_〉⋒〈*F*_2_, *A*_2_〉⋐_(*γ*,*δ*)_〈*G*_1_, *A*_1_〉⋒〈*G*_2_, *A*_2_〉 if *A*_1_∩*A*_2_ ≠ *∅*.

## 4. (∈_*γ*_, ∈_*γ*_∨*q*_*δ*_)-Fuzzy Soft (Invertible) *n*-Ary Subhypergroups over a Commutative *n*-Ary Hypergroup

In this section, we will introduce and investigate the concept of (∈_*γ*_, ∈_*γ*_∨*q*_*δ*_)-fuzzy soft *n*-ary subhypergroups over a commutative *n*-ary hypergroup. Let us start by giving the following concept.

**Definition 14 .**

Let 〈*F*, *A*〉 ∈ *FS*(*H*, *E*). Then 〈*F*, *A*〉 is called (∈_*γ*_, ∈_*γ*_∨*q*_*δ*_)-*fuzzy soft n*-*ary subhypergroup* over *H* if it satisfies the following conditions:

(F1a) for all *x* ∈ *H* and *ε* ∈ *A*, max⁡{*F*(*ε*)(*e*), *γ*} ≥ min⁡{*F*(*ε*)(*x*), *δ*};
(F2a) for all *x*_1_^*n*^ ∈ *H* and *ε* ∈ *A*, max⁡{inf⁡_*x*∈*f*(*x*_1_^*n*^)_⁡*F*(*ε*)(*x*), *γ*} ≥ min⁡{*F*(*ε*)_*x*_1__^*x*_*n*_^, *δ*};
(F3a) for all *y*, *y*_1_^*n*−1^ ∈ *H* and *ε* ∈ *A*, there exists *x* ∈ *H* such that
  (10) $$\begin{array}{c} y\in f\left(x,y_{1}^{n-1}\right),\\ \max\left\{F\left(\varepsilon \right)\left(x\right),\gamma \right\}\geq \min\left\{F{\left(\varepsilon \right)}_{y_{1}}^{y_{n-1}},F\left(\varepsilon \right)\left(y\right),\delta \right\}. \end{array}$$

An (∈_*γ*_, ∈_*γ*_∨*q*_*δ*_)-fuzzy *n*-ary subhypergroup 〈*F*, *A*〉 over *H* is said to be* invertible* if it satisfies the following:

(F4a) for all *x*, *y* ∈ *H* and *ε* ∈ *A*,
  (11) max⁡{min⁡{F(y,F(ε),…,F(ε)︸n−1)(x),δ},γ} =max⁡{min⁡{F(x,F(ε),…,F(ε)︸n−1)(y),δ},γ}.

**Example 15 .**

Let *Z*_8_ be the residue class of (*Z*, +) modulo 8 with an *n*-ary hyperoperation *f* as follows:

(12) $$\begin{array}{c} f:Z_{8}^{n}⟶P^{*}\left(Z_{8}\right),\\ f\left(x_{1}^{n}\right)=\left\{m_{1}x_{1}+\cdots +m_{n}x_{n}\;|\;m_{1},\ldots ,m_{n}\in Z\right\}. \end{array}$$

Then, (*Z*_8_, *f*) is a commutative *n*-ary hypergroup. Let *E* = (0.2,0.6] and *r* ≥ 0.6. Define a fuzzy soft set 〈*F*, *A*〉 over *Z*_8_ by

(13) F(ε)(x)={rif  x=0,εif  x∈{2,4},0.2otherwise,

for all *ε* ∈ *E* and *x* ∈ *Z*_8_. Then 〈*F*, *E*〉 is an (∈_0.2_, ∈_0.2_∨*q*_0.6_)-fuzzy soft invertible *n*-ary subhypergroup over *Z*_8_.

Next let us first provide some auxiliary lemmas as follows.

**Lemma 16 .**

Let 〈*F*, *A*〉 ∈ *FS*(*H*, *E*). Then (F2a) holds if and only if one of the following conditions holds:

(F2b) $F\left(\underset{n}{\underset{︸}{\langle F,A\rangle ,\ldots ,\langle F,A\rangle }}\right){⋐}_{\left(\gamma ,\delta \right)}\langle F,A\rangle$;
(F2c) for all *x*_1_^*n*^ ∈ *H* and *ε* ∈ *A*, *F*(*F*(*ε*)(*x*_1_)_*x*_1__,…, *F*(*ε*)(*x*_*n*_)_*x*_*n*__)  ⊆∨*q*_(*γ*,*δ*)_ *F*(*ε*);
(F2d) for all *ε* ∈ *A* and *r*_*i*_*x*_*i*___∈_*γ*_ *F*(*ε*)  (1 ≤ *i* ≤ *n*), *F*(*r*_1_*x*_1___,…, *r*_*n*_*x*_*n*___)⊆∨*q*_(*γ*,*δ*)_ *F*(*ε*).

**Proof**

(F2a)⇒(F2b) Assume that (F2a) holds. Then, for any *x* ∈ *H* and *ε* ∈ *A*, we have

(14) min⁡{F(F(ε),…,F(ε)︸n)(x),δ} =min⁡{sup⁡x∈f(y1n)min⁡{F(ε)y1yn},δ} =sup⁡x∈f(y1n)min⁡{F(ε)y1yn,δ}≤max⁡{F(ε)(x),γ}

and so $F\left(\underset{n}{\underset{︸}{F\left(\varepsilon \right),\ldots ,F\left(\varepsilon \right)}}\right)\subseteq \vee q_{\left(\gamma ,\delta \right)}F\left(\varepsilon \right)$ by Lemma 2. Therefore, $F\left(\underset{n}{\underset{︸}{\langle F,A\rangle ,\ldots ,\langle F,A\rangle }}\right){⋐}_{\left(\gamma ,\delta \right)}\langle F,A\rangle$.

(F2b)⇒(F2c) Let *x*_1_^*n*^ ∈ *H* and *ε* ∈ *A*. Since *F*(*ε*)(*x*_*i*_)_*x*_*i*__∈_*γ*_ *F*(*ε*), for all 1 ≤ *i* ≤ *n*, we have

(15) F(F(ε)(x1)x1,…,F(ε)(xn)xn) ⊆F(F(ε),…,F(ε)︸n)⊆∨q(γ,δ)F(ε),

and so *F*(*F*(*ε*)(*x*_1_)_*x*_1__,…, *F*(*ε*)(*x*_*n*_)_*x*_*n*__)⊆∨*q*_(*γ*,*δ*)_ *F*(*ε*).

(F2c)⇔(F2d) This is straightforward.

(F2d)⇒(F2a) Let *ε* ∈ *A*, *x* ∈ *H*, and *x*_1_^*n*^ ∈ *H* be such that *x* ∈ *f*(*x*_1_^*n*^). Then, by Lemma 2 and (F2d), we have

(16) max⁡{F(ε)(x),γ} ≥min⁡{F(F(ε)(x1)x1,…,F(ε)(xn)xn)(x),δ} =min⁡{(min⁡{F(ε)x1xn}f(x1n))(x),δ} =min⁡{F(ε)x1xn,δ}.

This implies max⁡{inf⁡_*x*∈*f*(*x*_1_^*n*^)_⁡*F*(*ε*)(*x*), *γ*} ≥ min⁡{*F*(*ε*)_*x*_1__^*x*_*n*_^, *δ*} for all *x*_1_^*n*^ ∈ *H* and *ε* ∈ *A*. Hence, (F2a) holds.

**Lemma 17 .**

Let 〈*F*, *A*〉∈*FS*(*H*, *E*). Then, one has the following.

(1) (F3a) holds if and only if the following condition holds: (F3b) for all *y*, *y*_1_^*n*−1^ ∈ *H* and *ε* ∈ *A*, there exists *x* ∈ *H* such that
  (17) min⁡{F(ε)y1yn−1,F(ε)(y)}y ⊆∨q(γ,δ)F(F(ε)(x)x,F(ε)(y1)y1,…,F(ε)(yn−1)yn−1).
(2) If (F3a) holds, then the following condition holds:
(F3c) for all *y*_1_^*n*−1^ ∈ *H* and *ε* ∈ *A*,
  (18) F(ε)∩min⁡{F(ε)y1yn−1}H ⊆∨q(γ,δ)F(F(ε),F(ε)(y1)y1,…,F(ε)(yn−1)yn−1).

Moreover, (F3c) implies (F3a) if 〈*F*, *A*〉 has the sup-property or *H* is finite.

**Proof**

(F3a)⇒(F3b) Let *y*, *y*_1_^*n*−1^ ∈ *H* and *ε* ∈ *A*. By (F3a), there exists *x* ∈ *H* such that

(19) $$\begin{array}{c} y\in f\left(x,y_{1}^{n-1}\right),\\ \max\left\{F\left(\varepsilon \right)\left(x\right),\gamma \right\}\geq \min\left\{F{\left(\varepsilon \right)}_{y_{1}}^{y_{n-1}},F\left(\varepsilon \right)\left(y\right),\delta \right\}. \end{array}$$

This implies min⁡{max⁡{*F*(*ε*)_*y*_1__^*y*_*n*−1_^, *γ*},  max⁡{*F*(*ε*)(*x*), *γ*}} ≥ min⁡{*F*(*ε*)_*y*_1__^*y*_*n*−1_^, *F*(*ε*)(*y*), *δ*}, and so

(20) max⁡{F(F(ε)(x)x,F(ε)(y1)y1,…,F(ε)(yn−1)yn−1)(y),γ} =max⁡{(min⁡{F(ε)y1yn−1,F(ε)(x)}f(x,y1n−1))(y),γ} ≥min⁡{F(ε)y1yn−1,F(ε)(y),δ} =min⁡{(min⁡{F(ε)y1yn−1,F(ε)(y)})y(y),δ}.

It follows that min⁡{*F*(*ε*)_*y*_1__^*y*_*n*−1_^,*F*(*ε*)(*y*)}_*y*_⊆∨*q*_(*γ*,*δ*)_ *F*(*F*(*ε*)(*x*)_*x*_, *F*(*ε*)(*y*_1_)_*y*_1__,…,*F*(*ε*)(*y*_*n*−1_)_*y*_*n*−1__) and so (F3b) holds.

(F3b)⇒(F3a) Let *y*, *y*_1_^*n*−1^ ∈ *H*. By (F3b), there exists *x* ∈ *H* such that

(21) min⁡{F(ε)y1yn−1,F(ε)(y)}y ⊆∨q(γ,δ)F(F(ε)(x)x,F(ε)(y1)y1,…,F(ε)(yn−1)yn−1).

It follows from Lemma 2 that

(22) max⁡{(min⁡{F(ε)y1yn−1,F(ε)(x)}f(x,y1n−1))(y),γ} =max⁡{F(F(ε)(x)x,F(ε)(y1)y1,…,F(ε)(yn−1)yn−1)×(y),γ} ≥min⁡{(min⁡⁡{F(ε)y1yn−1,F(ε)(y)})y(y),δ} =min⁡{F(ε)y1yn−1,F(ε)(y),δ}.

This implies *y* ∈ *f*(*x*, *y*_1_^*n*−1^) and max⁡⁡{min⁡⁡{*F*(*ε*)_*y*_1__^*y*_*n*−1_^,*F*(*ε*)(*x*)},*γ*} ≥ min⁡⁡{*F*(*ε*)_*y*_1__^*y*_*n*−1_^, *F*(*ε*)(*y*),*δ*}. Hence,

(23) max⁡{F(ε)(x),γ}≥max⁡{min⁡{F(ε)y1yn−1,F(ε)(x)},γ}≥min⁡{F(ε)y1yn−1,F(ε)(y),δ}

and so (F3a) holds.

(F3a)⇒(F3c) This is straightforward by (F3b).

In the following, assume that 〈*F*, *A*〉 has the sup-property or *H* is finite. We prove (F3c)⇒(F3a). Let *y*_1_^*n*−1^ ∈ *H*. If, for any *x* ∈ *H* such that *y* ∈ *f*(*x*, *y*_1_^*n*−1^), we have

(24) F(ε)(x)≤max⁡{F(ε)(x),γ}<min⁡{F(ε)y1yn−1,F(ε)(y),δ}≤F(ε)y1yn−1,

then since  〈*F*, *A*〉 has the sup-property or *H* is finite, we have

(25) max⁡{F(F(ε),F(ε)(y1)y1,…,F(ε)(yn−1)yn−1)(y),γ} =max⁡{sup⁡y∈f(x,y1n−1)min⁡{F(ε)(x),F(ε)y1yn−1},γ} =max⁡{sup⁡y∈f(x,y1n−1)F(ε)(x),γ} =sup⁡y∈f(x,y1n−1)max⁡{F(ε)(x),γ}   <min⁡{F(ε)y1yn−1,F(ε)(y),δ} =min⁡{(F(ε)∩min⁡{F(ε)y1yn−1}H)(y),δ},

which contradicts *F*(*ε*)∩min⁡{*F*(*ε*)_*y*_1__^*y*_*n*−1_^}_*H*_⊆∨*q*_(*γ*,*δ*)_ *F*(*F*(*ε*), *F*(*ε*)(*y*_1_)_*y*_1__,…,*F*(*ε*)(*y*_*n*−1_)_*y*_*n*−1__) by Lemma 2. Hence (F3a) holds.

**Lemma 18 .**

Let 〈*F*, *A*〉∈*FS*(*H*, *E*). Then (F4a) holds if and only if the following condition holds:

(F4c) for all *x*, *y* ∈ *H*, *ε* ∈ *A*, and *r* ∈ (*γ*, 1], $r_{x}\in_{\gamma }F\left(r_{y},\underset{n-1}{\underset{︸}{F\left(\varepsilon \right),\ldots ,F\left(\varepsilon \right)}}\right)$ implies $r_{y}\;\in_{\gamma }\vee q_{\delta }F\left(r_{x},\underset{n-1}{\underset{︸}{F\left(\varepsilon \right),\ldots ,F\left(\varepsilon \right)}}\right)$.

**Proof**

(F4a)⇒(F4c) Let *x*, *y* ∈ *H* and *r* ∈ (*γ*, 1]. If $r_{y}\in_{\gamma }F\left(r_{x},\underset{n-1}{\underset{︸}{F\left(\varepsilon \right),\ldots ,F\left(\varepsilon \right)}}\right)$ but $r_{x}\bar{\in_{\gamma }\vee q_{\delta }}$ $F\left(r_{y},\underset{n-1}{\underset{︸}{F\left(\varepsilon \right),\ldots ,F\left(\varepsilon \right)}}\right)$. Then

(26) F(x,F(ε),…,F(ε)︸n−1)(y) ≥F(rx,F(ε),…,F(ε)︸n−1)≥r>γ,

but *F*(*r*_*y*_,$\underset{n-1}{\underset{︸}{F(\varepsilon ),\ldots ,F(\varepsilon )}})(x)<r$ and *F*(*r*_*y*_,$\underset{n-1}{\underset{︸}{F(\varepsilon ),\ldots ,F(\varepsilon )}})(x)+r\leq 2\delta$. Then

(27) min⁡{F(y,F(ε),…,F(ε)︸n−1)(x),r} =F(ry,F(ε),…,F(ε)︸n−1)(x)<r

gives $F(y,\underset{n-1}{\underset{︸}{\mu ,\ldots ,\mu }})\left(x\right)<r$ and so $2F(y,\underset{n-1}{\underset{︸}{F\left(\varepsilon \right),\ldots ,F\left(\varepsilon \right)}})\left(x\right)$ $<\min\{F(y,\underset{n-1}{\underset{︸}{F\left(\varepsilon \right),\ldots ,F\left(\varepsilon \right)}})\left(x\right),r\}+r=F(r_{y}$,$\underset{n-1}{\underset{︸}{F\left(\varepsilon \right),\ldots ,F\left(\varepsilon \right)}})\left(x\right)+r\leq 2\delta$; that is, *F*(*y*, $\underset{n-1}{\underset{︸}{F\left(\varepsilon \right),\ldots ,F\left(\varepsilon \right)}})\left(x\right)<\delta$. Thus we have $\max\{F(y,\underset{n-1}{\underset{︸}{F\left(\varepsilon \right),\ldots ,F\left(\varepsilon \right)}})\left(x\right),\gamma \}<\min\{F(x,\underset{n-1}{\underset{︸}{F\left(\varepsilon \right),\ldots ,F\left(\varepsilon \right)}})\left(y\right),\delta \}$, a contradiction. Hence (F4c) holds.

(F4c)⇒(F4a) Let *x*, *y* ∈ *H*. If there exists *r* such that $\max\left\{F\left(y,\underset{n-1}{\underset{︸}{F\left(\varepsilon \right),\ldots ,F\left(\varepsilon \right)}}\right)\left(x\right),\gamma \right\}<r<\min\left\{F\left(x,\underset{n-1}{\underset{︸}{F\left(\varepsilon \right),\ldots ,F\left(\varepsilon \right)}}\right)\left(y\right),\delta \right\}$, then

(28) F(ry,F(ε),…,F(ε)︸n−1)(x) =min⁡{F(y,F(ε),…,F(ε)︸n−1)(x),r}<r ≤min⁡{F(x,F(ε),…,F(ε)︸n−1)(y),r} =F(rx,F(ε),…,F(ε)︸n−1)(y)

and *γ* < *r* < *δ*. This implies that *r*_*y*_∈_*γ*_ *F*(*r*_*x*_,$\underset{n-1}{\underset{︸}{F\left(\varepsilon \right),\ldots ,F\left(\varepsilon \right)}})$ but $r_{x}\bar{\in_{\gamma }\vee q_{\delta }}\;F(r_{y}$,$\underset{n-1}{\underset{︸}{F\left(\varepsilon \right),\ldots ,F\left(\varepsilon \right)}})$, a contradiction. Hence max⁡⁡{*F*(*y*,$\underset{n-1}{\underset{︸}{F\left(\varepsilon \right),\ldots ,F\left(\varepsilon \right)}})\left(x\right)$,*γ*} ≥ min⁡⁡{*F*(*x*, $\underset{n-1}{\underset{︸}{F\left(\varepsilon \right),\ldots ,F\left(\varepsilon \right)}})\left(y\right)$,*δ*} and so max⁡⁡{min⁡⁡{*F*(*y*, $\underset{n-1}{\underset{︸}{F\left(\varepsilon \right),\ldots ,F\left(\varepsilon \right)}})\left(x\right)$,*δ*},*γ*} ≥ max⁡⁡{min⁡⁡{*F*(*x*, $\underset{n-1}{\underset{︸}{F\left(\varepsilon \right),\ldots ,F\left(\varepsilon \right)}})\left(y\right)$,*δ*},*γ*}. In a similar way, we have max⁡⁡{min⁡⁡{*F*(*x*,$\underset{n-1}{\underset{︸}{F\left(\varepsilon \right),\ldots ,F\left(\varepsilon \right)}})\left(y\right)$,*δ*},*γ*} ≥ max⁡⁡{min⁡⁡{*F*(*y*,$\underset{n-1}{\underset{︸}{F\left(\varepsilon \right),\ldots ,F\left(\varepsilon \right)}})\left(x\right)$,*δ*},*γ*}. Thus (F4a) holds.

For any fuzzy soft set 〈*F*, *A*〉 over *H*, *ε* ∈ *A*, and  *r* ∈ (*γ*, 1], denote  *F*(*ε*)_*r*_ = {*x* ∈ *H*∣*x*_*r*_∈_*γ*_ *F*(*ε*)}  , 〈*F*(*ε*)〉_*r*_ = {*x* ∈ *H*∣*x*_*r*_ *q*_*δ*_ *F*(*ε*)}, and [*F*(*ε*)]_*r*_ = {*x* ∈ *H*∣*x*_*r*_∈_*γ*_∨*q*_*δ*_ *F*(*ε*)}. The next theorem presents the relationships between (∈_*γ*_, ∈_*γ*_∨*q*_*δ*_)-fuzzy soft (invertible) *n*-ary subhypergroups over *H* and crisp (invertible) *n*-ary subhypergroups of *H*.

**Theorem 19 .**

Let 〈*F*, *A*〉∈*FS*(*H*, *E*). Then,

1. 〈*F*, *A*〉 is an (∈_*γ*_, ∈_*γ*_∨*q*_*δ*_)-fuzzy soft (invertible) *n*-ary subhypergroup over *H* if and only if nonempty subset *F*(*ε*)_*r*_ is an (invertible) *n*-ary subhypergroup of *H* for all *ε* ∈ *A* and *r* ∈ (*γ*, *δ*];
2. if 2*δ* = 1 + *γ*, then 〈*F*, *A*〉 is an (∈_*γ*_, ∈_*γ*_∨*q*_*δ*_)-fuzzy soft (invertible) *n*-ary subhypergroup over *H* if and only if nonempty subset 〈*F*(*ε*)〉_*r*_ is an (invertible) *n*-ary subhypergroup of *H* for all *ε* ∈ *A* and *r* ∈ (*δ*, 1];
3. 〈*F*, *A*〉 is an (∈_*γ*_, ∈_*γ*_∨*q*_*δ*_)-fuzzy soft (invertible) *n*-ary subhypergroup over *H* if and only if nonempty subset [*F*(*ε*)]_*r*_ is an (invertible) *n*-ary subhypergroup of *H* for all *ε* ∈ *A* and *r* ∈ (*γ*, min⁡{2*δ* − *γ*, 1}].

**Proof**

We only prove (2) and (3). (1) can be easily proved.

(2) Assume that 2*δ* = 1 + *γ*. Let 〈*F*, *A*〉 be an (∈_*γ*_, ∈_*γ*_∨*q*_*δ*_)-fuzzy soft *n*-ary subhypergroup over *H* and assume that 〈*F*(*ε*)〉_*r*_ ≠ *∅* for some *ε* ∈ *A* and *r* ∈ (*δ*, 1]. Let *x*_1_^*n*^ ∈ 〈*F*(*ε*)〉_*r*_. Then *r*_*x*_1__ *q*_*δ*_ *F*(*ε*),…, *r*_*x*_*n*__ *q*_*δ*_ *F*(*ε*); that is, *F*(*ε*)(*x*_1_) + *r* > 2*δ*,…, *F*(*ε*)(*x*_*n*_) + *r* > 2*δ*. Since 〈*F*, *A*〉 is an (∈_*γ*_, ∈_*γ*_∨*q*_*δ*_)-fuzzy *n*-ary subhypergroup over *H*, we have max⁡{*F*(*ε*)(*x*), *γ*} ≥ min⁡{*F*(*ε*)_*x*_1__^*x*_*n*_^, *δ*} for all *x* ∈ *f*(*x*_1_^*n*^). Hence, by *r* > *δ*,

(29) max⁡{F(ε)(x)+r,γ+r} =max⁡{F(ε)(x),γ}+r≥min⁡{F(ε)x1xn,δ}+r =min⁡{F(ε)(x1)+r,…,F(ε)(xn)+r,δ+r}>2δ.

From *r* ≤ 1 = 2*δ* − *γ*, that is, *r* + *γ* ≤ 2*δ*, we have *F*(*ε*)(*x*) + *r* > 2*δ* and so *x* ∈ 〈*F*(*ε*)〉_*r*_. Similarly we can show that *e* ∈ 〈*F*(*ε*)〉_*r*_ and, for all *y*, *y*_1_^*n*−1^ ∈ 〈*F*(*ε*)〉_*r*_, there exists *x* ∈ 〈*F*(*ε*)〉_*r*_ such that *y* ∈ *f*(*x*, *y*_1_^*n*−1^). Therefore, 〈*F*(*ε*)〉_*r*_ is an *n*-ary hypergroup of *H*.

Conversely, assume that the given condition holds. Let *x*_1_^*n*^ ∈ *H* and *ε* ∈ *A*. If there exists *x* ∈ *f*(*x*_1_^*n*^) such that max⁡{*F*(*ε*)(*x*), *γ*} < min⁡{*F*(*ε*)_*x*_1__^*x*_*n*_^, *δ*}, take *r* = 2*δ* − max⁡{*F*(*ε*)(*x*), *γ*}. Then *r* ∈ (*δ*, 1], *F*(*ε*)(*x*) ≤ 2*δ* − *r*, *F*(*ε*)(*x*_1_) > max⁡{*G*(*ε*)(*x*), *γ*} = 2*δ* − *r*,…, *F*(*ε*)(*x*_*n*_) > max⁡{*G*(*ε*)(*x*), *γ*} = 2*δ* − *r*, that is, *x*_1_^*n*^ ∈ 〈*F*(*ε*)〉_*r*_, but  *x* ∉ 〈*F*(*ε*)〉_*r*_, a contradiction. Hence max⁡{*F*(*ε*)(*x*), *γ*} ≥ min⁡{*F*(*ε*)_*x*_1__^*x*_*n*_^, *δ*} and so max⁡{inf⁡_*x*∈*f*(*x*_1_^*n*^)_⁡*F*(*ε*)(*x*), *γ*} ≥ min⁡{*F*(*ε*)_*x*_1__^*x*_*n*_^, *δ*}. Similarly we can show that conditions (F1a) and (F3a) hold. Therefore, 〈*F*, *A*〉 is an (∈_*γ*_, ∈_*γ*_∨*q*_*δ*_)-fuzzy soft *n*-ary subhypergroup over *H*.

(3) Let 〈*F*, *A*〉 be an (∈_*γ*_, ∈_*γ*_∨*q*_*δ*_)-fuzzy soft *n*-ary subhypergroup over *H* and assume that [*F*(*ε*)]_*r*_ ≠ *∅* for some *ε* ∈ *A* and *r* ∈ (*γ*, min⁡{2*δ* − *γ*, 1}]. Let *x*_1_^*n*^ ∈ [*F*(*ε*)]_*r*_. Then *r*_*x*_1__∈_*γ*_∨*q*_*δ*_ *F*(*ε*),…, *r*_*x*_*n*__∈_*γ*_∨*q*_*δ*_ *F*(*ε*); that is, *F*(*ε*)(*x*_1_) ≥ *r* > *γ* or *F*(*ε*)(*x*_1_) > 2*δ* − *r* ≥ 2*δ* − (2*δ* − *γ*) = *γ*,…, *F*(*ε*)(*x*_*n*_) ≥ *r* > *γ* or *F*(*ε*)(*x*_*n*_) > 2*δ* − *r* ≥ 2*δ* − (2*δ* − *γ*) = *γ*. Since 〈*F*, *A*〉 is an (∈_*γ*_, ∈_*γ*_∨*q*_*δ*_)-fuzzy *n*-ary subhypergroup over *H*, we have max⁡{*F*(*ε*)(*x*), *γ*} ≥ min⁡{*F*(*ε*)_*x*_1__^*x*_*n*_^, *δ*} for all *x* ∈ *f*(*x*_1_^*n*^) and so *F*(*ε*)(*x*) ≥ min⁡{*F*(*ε*)_*x*_1__^*x*_*n*_^, *δ*} since *γ* < min⁡{*F*(*ε*)_*x*_1__^*x*_*n*_^, *δ*} in any case. Now we consider the following cases.

*Case  1 *(*r* ∈ (*γ*, *δ*]). In this case, 2*δ* − *r* ≥ *δ* ≥ *r*.

1. If *F*(*ε*)(*x*_1_) ≥ *r* or ⋯ or *F*(*ε*)(*x*_*n*_) ≥ *r*, then *F*(*ε*)(*x*) ≥ min⁡{*F*(*ε*)_*x*_1__^*x*_*n*_^, *δ*} ≥ *r*. Hence *z*_*r*_∈_*γ*_ *F*(*ε*).
2. If *F*(*ε*)(*x*_*i*_) + *r* > 2*δ*, for all 1 ≤ *i* ≤ *n*, then *F*(*ε*)(*x*) ≥ min⁡{*F*(*ε*)_*x*_1__^*x*_*n*_^, *δ*} = *δ* ≥ *r*. Hence *z*_*r*_∈_*γ*_ *F*(*ε*).

*Case  2 *(*r* ∈ (*δ*, min⁡{2*δ* − *γ*, 1}]). In this case, *r* > *δ* > 2*δ* − *r*.

1. If *F*(*ε*)(*x*_*i*_) ≥ *r*, for all 1 ≤ *i* ≤ *n*, then *F*(*ε*)(*x*) ≥ min⁡{*F*(*ε*)_*x*_1__^*x*_*n*_^, *δ*} = *δ* > 2*δ* − *r*. Hence *z*_*r*_ *q*_*δ*_ *F*(*ε*).
2. If *F*(*ε*)(*x*_1_) + *r* > 2*δ* or ⋯ or *F*(*ε*)(*x*_*n*_) + *r* > 2*δ*, then *F*(*ε*)(*x*) ≥ min⁡{*F*(*ε*)_*x*_1__^*x*_*n*_^, *δ*} > 2*δ* − *r*. Hence *z*_*r*_ *q*_*δ*_ *F*(*ε*).

Thus, in any case, *x*_*r*_∈_*γ*_∨*q*_*δ*_ *F*(*ε*); that is, *x* ∈ [*F*(*ε*)]_*r*_. Similarly, we can show that *e* ∈ [*F*(*ε*)]_*r*_ and, for all *y*, *y*_1_^*n*−1^ ∈ [*F*(*ε*)]_*r*_, there exists *x* ∈ [*F*(*ε*)]_*r*_ such that *y* ∈ *f*(*x*, *y*_1_^*n*−1^). Therefore, [*F*(*ε*)]_*r*_ is an *n*-ary hypergroup of *H*.

Conversely, assume that the given condition holds. Let *x*_1_^*n*^ ∈ *H* and *ε* ∈ *A*. If there exists *x* ∈ *f*(*x*_1_^*n*^) such that max⁡{*F*(*ε*)(*x*), *γ*} < *r* = min⁡{*F*(*ε*)_*x*_1__^*x*_*n*_^, *δ*}, then *F*(*ε*)(*x*_1_) ≥ *r* > *γ*,…, *F*(*ε*)(*x*_*n*_) ≥ *r* > *γ*, *F*(*ε*)(*x*) < *r*, and *F*(*ε*)(*x*) + *r* < 2*r* ≤ 2*δ*, that is, (*x*_1_)_*r*_∈_*γ*_ *F*(*ε*),…, (*x*_*n*_)_*r*_∈_*γ*_ *F*(*ε*), but $x_{r}\bar{\in_{\gamma }\vee q_{\delta }}F\left(\varepsilon \right)$, that is, *x*_1_^*n*^ ∈ [*F*(*ε*)]_*r*_, but *x* ∉ [*F*(*ε*)]_*r*_, a contradiction. Hence max⁡{*F*(*ε*)(*x*), *γ*} ≥ min⁡{*F*(*ε*)_*x*_1__^*x*_*n*_^, *δ*} and so max⁡{inf⁡_*x*∈*f*(*x*_1_^*n*^)_⁡*F*(*ε*)(*x*), *γ*} ≥ min⁡{*F*(*ε*)_*x*_1__^*x*_*n*_^, *δ*}. Similarly, we can show that conditions (F1a) and (F3a) hold. Therefore, 〈*F*, *A*〉 is an (∈_*γ*_, ∈_*γ*_∨*q*_*δ*_)-fuzzy soft *n*-ary subhypergroup over *H*.

As a direct consequence of Theorem 19, we have the following results.

**Corollary 20 .**

Let *γ*, *γ*′, *δ*, *δ*′ ∈ [0,1] be such that *γ* < *δ*, *γ*′ < *δ*′, *γ* < *γ*′, and  *δ*′ < *δ*. Then every (∈_*γ*_, ∈_*γ*_∨*q*_*δ*_)-fuzzy (invertible) *n*-ary soft subhypergroup over *H* is an (∈_*γ*′_, ∈_*γ*′_∨*q*_*δ*′_)-fuzzy soft (invertible) *n*-ary subhypergroup over *H*.

**Corollary 21 .**

Let *P* ∈ P*(*H*). Then *P* is an (invertible) *n*-ary subhypergroup of *H* if and only if Σ(*P*, *A*, *r*) is an (∈_*γ*_, ∈_*γ*_∨*q*_*δ*_)-fuzzy soft (invertible) *n*-ary subhypergroup over *H* for any *A*⊆*E* and *r* ≥ *δ*.

**Lemma 22 .**

Let 〈*F*, *A*〉 ∈ *FS*(*H*, *E*). Then

(30) 〈F,A〉⋐(γ,δ)F(〈1e~,A〉,…,〈1e~,A〉︸i−1,〈F,A〉,       〈1e~,A〉,…,〈1e~,A〉︸n−i)

for all 1 ≤ *i* ≤ *n*.

**Proof**

It is straightforward.

**Lemma 23 .**

Let 〈*F*, *A*〉 be an (∈_*γ*_, ∈_*γ*_∨*q*_*δ*_)-fuzzy soft *n*-ary subhypergroup over *H*. Then

1. $F\left(\underset{i}{\underset{︸}{\langle F,A\rangle ,\ldots ,\langle F,A\rangle }},\underset{n-i}{\underset{︸}{\langle \tilde{1_{e}},A\rangle ,\ldots ,\langle \tilde{1_{e}},A\rangle }}\right){≍}_{\left(\gamma ,\delta \right)}\langle F,A\rangle$ for all 1 ≤ *i* ≤ *n*;
2. $F(\langle \tilde{1_{x}},A\rangle ,\underset{i}{\underset{︸}{\langle F,A\rangle ,\ldots ,\langle F,A\rangle }}$, 〈1e~,A〉,…,〈1e~,A〉︸n-i-1)≍(γ,δ)F(〈F,A〉,…,〈F,A〉︸n-1〈1x~,A〉, $\underset{n-1}{\underset{︸}{\langle F,A\rangle ,\ldots ,\langle F,A\rangle }})$ for all *x* ∈ *H* and 1 ≤ *i* ≤ *n*.

**Proof**

(1) Let 〈*F*, *A*〉 be an (∈_*γ*_, ∈_*γ*_∨*q*_*δ*_)-fuzzy soft *n*-ary subhypergroup over *H*, *ε* ∈ *A*, and 1 ≤ *i* ≤ *n*. Since max⁡{*F*(*ε*)(*e*), *γ*} ≥ min⁡{*F*(*ε*)(*x*), *δ*}, for all *x* ∈ *H*, we have

(31) F(ε)⊆∨q(γ,δ)F(F(ε),1e,…,1e︸n−1)⊆∨q(γ,δ)F(F(ε),…,F(ε)︸i,1e,…,1e︸n−i)⊆∨q(γ,δ)F(F(ε),…,F(ε)︸n)⊆∨q(γ,δ)F(ε).

Therefore, $F\left(F\left(\varepsilon \right),\underset{n-1}{\underset{︸}{1_{e},\ldots ,1_{e}}}\right){=}_{\left(\gamma ,\delta \right)}F\left(\varepsilon \right)$ and so $F\left(\underset{i}{\underset{︸}{\langle F,A\rangle ,\ldots ,\langle F,A\rangle }},\underset{n-i}{\underset{︸}{\langle \tilde{1_{e}},A\rangle ,\ldots ,\langle \tilde{1_{e}},A\rangle }}\right){≍}_{\left(\gamma ,\delta \right)}\langle F,A\rangle$.

(2) Let *x* ∈ *H* and 1 ≤ *i* ≤ *n*. By (1), we have

(32) F(〈1x~,A〉,〈F,A〉,…,〈F,A〉︸n−1) ≍(γ,δ)F(〈1x~,A〉,〈F,A〉,…,〈F,A〉︸n−2),  F(〈F,A〉,〈1e~,A〉,…,〈1e~,A〉︸n−1) ≍(γ,δ)F(〈1x~,A〉,〈F,A〉,…,〈F,A〉︸i−1,  F(〈F,A〉,…,〈F,A〉︸n−i,〈1e~,A〉,…,〈1e~,A〉︸i),〈1e~,A〉,…,〈1e~,A〉︸n−i−1) ≍(γ,δ)F(〈1x~,A〉,〈F,A〉,…,〈F,A〉︸i−1,  〈F,A〉,〈1e~,A〉,…,〈1e~,A〉︸n−i−1) ≍(γ,δ)F(〈1x~,A〉,〈F,A〉,…,〈F,A〉︸i, 〈1e~,A〉,…,〈1e~,A〉︸n−i−1).

This completes the proof.

**Lemma 24 .**

Let 〈*F*, *A*〉 be an (∈_*γ*_, ∈_*γ*_∨*q*_*δ*_)-fuzzy soft *n*-ary subhypergroup over *H*. Then 〈*F*, *A*〉 is invertible if and only if

(33) max⁡{min⁡{F(x,F(ε),…,F(ε)︸i,e∗)(y),δ},γ} =max⁡{min⁡{F(y,F(ε),…,F(ε)︸i,e∗)(x),δ},γ}

for all *x*, *y* ∈ *H*, *ε* ∈ *A*, and 1 ≤ i ≤ n. Proof

It is straightforward by Lemmas 18 and 23.

**Lemma 25 .**

Let 〈*F*, *A*〉,〈*F*_1_, *A*〉,…, 〈*F*_*i*_, *A*〉  (2 ≤ *i* ≤ *n*) be (∈_*γ*_, ∈_*γ*_∨*q*_*δ*_)-fuzzy soft invertible *n*-ary subhypergroups over *H*. Then

1. max⁡{min⁡{*F*(*x*, *F*_1_^*i*^(*ε*), *e*∗)(*y*), *δ*}, *γ*} = max⁡⁡{min⁡⁡{*F*(*y*,*F*_1_^*i*^(*ε*), *e*∗)(*x*),*δ*},*γ*}, for all *x*, *y* ∈ *H* and *ε* ∈ *A*;
2. for all  *x*, *y*, *y*_1_^*n*−1^ ∈ *H* such that *y* ∈ *f*(*x*, *y*_1_^*n*−1^) and *ε* ∈ *A*, one has max⁡{*F*(*ε*)(*x*), *γ*} ≥ min⁡{*F*(*ε*)_*y*_1__^*y*_*n*−1_^, *F*(*ε*)(*y*), *δ*}.

**Proof**

(1) Let *x*, *y* ∈ *H* and *ε* ∈ *A*. Then, by Lemma 23, we have

(34) F(x,F1i(ε),e∗)=F(x,F1(ε),F2(ε),F3i(ε),e∗) ≍(γ,δ)F(x,F1(ε),F(e,…,e︸n−2,F2(ε),e),F3i(ε),e∗) ≍(γ,δ)F(F(x,F1(ε),e∗),F2(ε),e,F3i(ε),e∗) ≍(γ,δ)F(F(x,F1(ε),e∗),F2i(ε),e∗) ≍(γ,δ)F(F(x,F1(ε),e∗),F(F2i(ε),e∗),e∗).

It follows that

(35) max⁡{min⁡⁡{F(x,F1i(ε),e∗)(y),δ},γ} ≥min⁡⁡{max⁡{F(F(x,F1(ε),e∗),F(F2i(ε),e∗),e∗)(y),γ},δ} =min⁡{max⁡{sup⁡y∈f(a1,a2,e∗)min⁡{F(x,F1(ε),e∗)(a1),F(F2i(ε),e∗)(a2)},γ},δ} =sup⁡z1∈Hmin⁡{max⁡{min⁡{F(x,F1(ε),e∗)(z1),F(z1,F2i(ε),e∗)(y)},γ},δ} =sup⁡z1,z2∈Hmin⁡⁡{max⁡{min⁡{F(x,F1(ε),e∗)(z1),F(z1,F2(ε),e∗)(z2),F(z2,F3i(ε),e∗)(y)},γ},δ} =⋯ =sup⁡z1i∈Hmin⁡{max⁡{min⁡{F(x,F1(ε),e∗)(z1),F(z1,F2(ε),e∗)(z2),…,F(zi−1,Fi(ε),e∗)(y)},γ},δ} ≥sup⁡z1i∈Hmin⁡{F(z1,F1(ε),e∗)(x),F(z2,F2(ε),e∗)(z1),…,F(y,Fi(ε),e∗)(zi−1),δ} =min⁡{F(y,Fi(ε),Fi−1(ε),…,F1(ε),e∗)(x),δ} =min⁡⁡{F(y,F1i(ε),e∗)(x),δ}.

This implies max⁡{min⁡{*F*(*x*, *F*_1_^*i*^(*ε*), *e*∗)(*y*), *δ*}, *γ*} ≥ max⁡{min⁡⁡{*F*(*y*, *F*_1_^*i*^(*ε*), *e*∗)(*x*), *δ*}, *γ*}. In a similar way, we have max⁡⁡{min⁡⁡{*F*(*y*, *F*_1_^*i*^(*ε*), *e*∗)(*x*), *δ*}, *γ*} ≥ max⁡⁡{min⁡⁡{*F*(*x*, *F*_1_^*i*^(*ε*), *e*∗)(*y*), *δ*}, *γ*}. Hence max⁡⁡{min⁡⁡{*F*(*x*, *F*_1_^*i*^(*ε*), *e*∗)(*y*), *δ*}, *γ*} = max⁡⁡{min⁡⁡{*F*_1_^*i*^(*y*, *F*(*ε*), *e*∗)(*x*), *δ*}, *γ*}.

(2) Let *x*, *y*, *y*_1_^*n*−1^ ∈ *H* be such that *y* ∈ *f*(*x*, *y*_1_^*n*−1^) and *ε* ∈ *A*. Then $F\left(x,\underset{n-1}{\underset{︸}{F\left(\varepsilon \right),\ldots ,F\left(\varepsilon \right)}}\right)\left(y\right)\geq \min\left\{F{\left(\varepsilon \right)}_{y_{1}}^{y_{n-1}}\right\}\;$. Thus, we have

(36) max⁡{F(ε)(x),γ} ≥max⁡{min⁡{F(F(ε),…,F(ε)︸n)(x),δ},γ} ≥max⁡{min⁡{F(ε)(y),F(y,F(ε),…,F(ε)︸n−1)(x),       δ},γ} ≥min⁡{F(ε)(y),max⁡{F(y,F(ε),…,F(ε)︸n−1)(x),           γ},δ} ≥min⁡{F(ε)(y),F(x,F(ε),…,F(ε)︸n−1)(y),δ} ≥min⁡{F(ε)(y),F(ε)y1yn−1,δ}.

This completes the proof.

**Theorem 26 .**

Let 〈*F*_*k*_, *A*〉 be (∈_*γ*_, ∈_*γ*_∨*q*_*δ*_)-fuzzy soft invertible *n*-ary subhypergroup over *H* for all 1 ≤ *k* ≤ *n* and 2 ≤ *i* ≤ *n*. Then $F\left(\langle F_{1},A\rangle ,\ldots ,\langle F_{i},A\rangle ,\underset{n-i}{\underset{︸}{\langle \tilde{1_{e}},A\rangle ,\ldots ,\langle \tilde{1_{e}},A\rangle }}\right)$ is an (∈_*γ*_, ∈_*γ*_∨*q*_*δ*_)-fuzzy soft invertible *n*-ary subhypergroup over *H*.

**Proof**

Since each 〈*F*_*k*_, *A*〉 is an (∈_*γ*_, ∈_*γ*_∨*q*_*δ*_)-fuzzy soft invertible *n*-ary subhypergroup over *H* for all  1 ≤ *k* ≤ *n*, let *ε* ∈ *A*. It is clear that max⁡⁡{*F*(*F*_1_^*i*^(*ε*),*e*∗)(*e*),*γ*} ≥ min⁡⁡{*F*(*F*_1_^*i*^(*ε*),*e*∗)(*x*),*δ*} for all *x* ∈ *H*. From Lemma 23, for any 2 ≤ *j* ≤ *n*, we have

(37) F(F(F1i(ε),e∗),…,F(F1i(ε),e∗)︸j,e∗) =F(F(F1(ε),…,F1(ε)︸j,e∗),…,F(Fi(ε),…,Fi(ε)︸j,e∗),e∗) ≍(γ,δ)F(F1i(ε),e∗).

In particular, we have

(38) F(F(F1i(ε),e∗),…,F(F1i(ε),e∗)︸n) ≍(γ,δ)F(F1i(ε),e∗).

Now we show that

(39) max⁡{min⁡{F(x,F(F1i(ε),e∗),…,F(F1i(ε),e∗)︸n−1)(y),δ},γ}  =max⁡{min⁡{F(y,F(F1i(ε),e∗),…,F(F1i(ε),e∗)︸n−1)(x),δ},γ}

for all *x*, *y* ∈ *H*; that is, *F*(*F*_1_^*i*^(*ε*), *e*∗) is invertible. In fact, by Lemmas 23 and 25(1), we have

(40) max⁡{min⁡{F(x,F(F1i(ε),e∗),…,F(F1i(ε),e∗)︸n−1)(y),δ},γ}  =max⁡{min⁡{F(x,F1i(ε),e∗)(y),δ},γ}  =max⁡{min⁡{F(y,F1i(ε),e∗)(x),δ},γ}=max⁡{min⁡{F(y,F(F1i(ε),e∗),…,F(F1i(ε),e∗)︸n−1)(x),δ},γ}.

Next, let *x*, *y*, *y*_1_^*n*−1^ ∈ *H* be such that *y* ∈ *f*(*x*, *y*_1_^*n*−1^). Then by Lemma 25(2), we have max⁡{*F*(*F*_1_^*i*^(*ε*), *e*∗)(*x*), *γ*} ≥ min⁡{*F*(*F*_1_^*i*^(*ε*),*e*∗)_*y*_1__^*y*_*n*−1_^, *F*(*F*_1_^*i*^(*ε*), *e*∗)(*y*), *δ*}.

Summing up the above arguments, $F\left(\langle F_{1},A\rangle ,\ldots ,\langle F_{i},A\rangle ,\underset{n-i}{\underset{︸}{\langle \tilde{1_{e}},A\rangle ,\ldots ,\langle \tilde{1_{e}},A\rangle }}\right)$ is an (∈_*γ*_, ∈_*γ*_∨*q*_*δ*_)-fuzzy soft invertible *n*-ary subhypergroup over *H*.

**Theorem 27 .**

Let 〈*F*, *A*〉 and 〈*G*, *B*〉 be two (∈_*γ*_, ∈_*γ*_∨*q*_*δ*_)-fuzzy soft invertible *n*-ary subhypergroups over *H*. Then both $\langle F,A\rangle \tilde{\cap }\langle G,B\rangle$ and 〈*F*, *A*〉⋒〈*G*, *B*〉 are (∈_*γ*_, ∈_*γ*_∨*q*_*δ*_)-fuzzy soft *n*-ary subhypergroups over *H*.

**Proof**

Let 〈*F*, *A*〉 and 〈*G*, *B*〉 be (∈_*γ*_, ∈_*γ*_∨*q*_*δ*_)-fuzzy soft invertible *n*-ary subhypergroups over *H* and $\langle F,A\rangle \tilde{\cap }\langle G,B\rangle =\langle L,A\cup B\rangle$. Then, for any *ε* ∈ *A* ∪ *B*, we consider the following cases.

*Case  1 *(*ε* ∈ *A* − *B*). In this case, *L*(*ε*) = *F*(*ε*). It follows that *L*(*ε*) satisfies conditions (F1a), (F2a), and (F3a) since 〈*F*, *A*〉 is an (∈_*γ*_, ∈_*γ*_∨*q*_*δ*_)-fuzzy soft invertible *n*-ary subhypergroup over *S*.

*Case  2 *(*ε* ∈ *B* − *A*). In this case, *L*(*ε*) = *G*(*ε*). It follows that *L*(*ε*) satisfies conditions (F1a), (F2a), and (F3a) since 〈*G*, *B*〉 is an (∈_*γ*_, ∈_*γ*_∨*q*_*δ*_)-fuzzy soft invertible *n*-ary subhypergroup over *S*.

*Case  3 *(*ε* ∈ *A*∩*B*). In this case, *L*(*ε*) = *F*(*ε*)∩*G*(*ε*). Now we show that *F*(*ε*)∩*G*(*ε*) satisfies conditions (F1a), (F2a), and (F3a).

(1) For any *x* ∈ *H*, we have
  (41) max⁡{(F(ε)∩G(ε))(e),γ} =max⁡{min⁡{F(ε)(e),G(ε)(e)},γ} =min⁡{max⁡{F(ε)(e),γ},max⁡{G(ε)(e),γ}} ≥min⁡{F(ε)(x),G(ε)(x),δ} =min⁡{(F(ε)∩G(ε))(x),δ}.
(2) Let *x*, *x*_1_^*n*^ ∈ *H* be such that *x* ∈ *f*(*x*_1_^*n*^). Then
  (42) max⁡{(F(ε)∩G(ε))(x),γ} =max⁡{min⁡{F(ε)(x),G(ε)(x)},γ} =min⁡{max⁡{F(ε)(x),γ},max⁡{G(ε)(x),γ}} ≥min⁡{F(ε)x1xn,G(ε)x1xn,δ} =min⁡{(F(ε)∩G(ε))x1xn,δ}.

It follows that max⁡{inf⁡_*x*∈*f*(*x*_1_^*n*^)_⁡(*F*(*ε*)∩*G*(*ε*))(*x*), *γ*} ≥ min⁡{(*F*(*ε*)∩*G*(*ε*))_*x*_1__^*x*_*n*_^, *δ*}.

(3) Let *x*, *y*, *y*_1_^*n*−1^ ∈ *H* be such that *y* ∈ *f*(*x*, *y*_1_^*n*−1^). Analogous to (2), we have
  (43) max⁡{(F(ε)∩G(ε))(x),γ} ≥min⁡{(F(ε)∩G(ε))y1yn−1,(F(ε)∩G(ε))(y),δ}.

Therefore, $\langle F,A\rangle \tilde{\cap }\langle G,B\rangle$ is an (∈_*γ*_, ∈_*γ*_∨*q*_*δ*_)-fuzzy soft *n*-ary subhypergroup over *H*. The case for 〈*F*, *A*〉⋒〈*G*, *B*〉 can be similarly proved.

## 5. The Homomorphism of (∈_*γ*_, ∈_*γ*_∨*q*_*δ*_)-Fuzzy Soft (Invertible) Subhypergroups

In this section, we consider the homomorphism properties of (∈_*γ*_, ∈_*γ*_∨*q*_*δ*_)-fuzzy soft (invertible) subhypergroups. Let us first introduce the following definition.

**Definition 28 .**

Let *FS*(*U*, *E*) and *FS*(*U*′, *E*′) be two fuzzy soft classes, and let *φ* : *U* → *U*′ and *ϕ* : *E* → *E*′ be mappings. Define a mapping (*φ*, *ϕ*) : *FS*(*U*, *E*) → *FS*(*U*′, *E*′) by the following: for 〈*F*, *A*〉 ∈ *FS*(*U*, *E*), the* image* of 〈*F*, *A*〉 under (*φ*, *ϕ*), denoted by (*φ*, *ϕ*)〈*F*, *A*〉 = 〈*φ*(*F*), *ϕ*(*A*)〉, is a fuzzy soft set in *FS*(*U*′, *E*′) given by

(44) φ(F)(ε′)(x′) ={sup⁡ε∈ϕ−1(ε′)∩A,x∈φ−1(x′)F(ε)(x)if  φ−1(x′)≠∅,0otherwise,

for all *ε*′ ∈ *ϕ*(*A*) and *x*′ ∈ *U*′. For 〈*F*′, *A*′〉 ∈ *FS*(*U*′, *E*′), the* inverse image* of 〈*F*′, *A*′〉 under (*φ*, *ϕ*), denoted by (*φ*,*ϕ*)^−1^〈*F*′, *A*′〉 = 〈*φ* ^−1^(*F*′), *ϕ* ^−1^(*A*′)〉, is a fuzzy soft set in *FS*(*U*, *E*) given by

(45) $$\begin{array}{c} \varphi^{-1}\left(F^{\prime }\right)\left(\varepsilon \right)\left(x\right)=F^{\prime }\left(\phi \left(\varepsilon \right)\right)\left(\varphi \left(x\right)\right) \end{array}$$

for all *ε* ∈ *ϕ* ^−1^(*B*) and *x* ∈ *U*.

The following results can be easily deduced.

**Lemma 29 .**

Let 〈*F*, *A*〉, 〈*G*, *B*〉∈*FS*(*U*, *E*) and *φ* : *U* → *U*′ and *ϕ* : *E* → *E*′ be two mappings. Then

1. 〈*F*, *A*〉⋐(*φ*,*ϕ*)^−1^((*φ*, *ϕ*)〈*F*, *A*〉) and 〈*F*, *A*〉 = (*φ*,*ϕ*)^−1^((*φ*, *ϕ*)〈*F*, *A*〉) if both *φ* and *ϕ* are injective;
2. (*φ*, *ϕ*)(〈*F*, *A*〉⋒〈*G*, *B*〉)⋐(*φ*, *ϕ*)〈*F*, *A*〉⋒(*φ*, *ϕ*)〈*G*, *B*〉;
3. $\left(\varphi ,\phi \right)\left(\langle F,A\rangle \tilde{\cap }\langle G,B\rangle \right)⋐\left(\varphi ,\phi \right)\langle F,A\rangle \tilde{\cap }\left(\varphi ,\phi \right)\langle G,B\rangle$ if *ϕ* is injective;
4. if 〈*F*, *A*〉⋐_(*γ*,*δ*)_〈*G*, *B*〉, then (*φ*, *ϕ*)〈*F*, *A*〉⋐_(*γ*,*δ*)_(*φ*, *ϕ*)〈*G*, *B*〉.

**Lemma 30 .**

Let 〈*F*′, *A*′〉, 〈*G*′, *B*′〉 ∈ *FS*(*U*′, *E*′) and *φ* : *U* → *U*′ and *ϕ* : *E* → *E*′ be two mappings. Then

1. (*φ*, *ϕ*)((*φ*,*ϕ*)^−1^〈*F*′, *A*′〉)⋐〈*F*′, *A*′〉 and (*φ*, *ϕ*)((*φ*,*ϕ*)^−1^〈*F*′, *A*′〉) = 〈*F*′, *A*′〉 if both *φ* and *ϕ* are surjective;
2. (*φ*,*ϕ*)^−1^(〈*F*′, *A*′〉⋒〈*G*′, *B*′〉) = (*φ*,*ϕ*)^−1^〈*F*′, *A*′〉⋒(*φ*,*ϕ*)^−1^〈*G*′, *B*′〉;
3. ${\left(\varphi ,\phi \right)}^{-1}\left(\langle F^{\prime },A^{\prime }\rangle \tilde{\cap }\langle G^{\prime },B^{\prime }\rangle \right)={\left(\varphi ,\phi \right)}^{-1}\langle F^{\prime },A^{\prime }\rangle \tilde{\cap }{\left(\varphi ,\phi \right)}^{-1}\langle G^{\prime },B^{\prime }\rangle \;$;
4. if 〈*F*′, *A*′〉⋐_(*γ*,*δ*)_〈*G*′, *B*′〉, then (*φ*,*ϕ*)^−1^〈*F*′, *A*′〉⋐_(*γ*,*δ*)_(*φ*,*ϕ*)^−1^〈*G*′, *B*′〉.

**Definition 31 .**

Let (*H*, *f*) and (*H*′, *f*′) be two *n*-ary hypergroups with identities *e* and *e*′, respectively, and *φ* a mapping from *H* to *H*′. Then, *φ* is called a* homomorphism* if *φ*(*e*) = *e*′ and *φ*(*f*(*x*_1_^*n*^)) = *f*′(*φ*(*x*_1_),…, *φ*(*x*_*n*_)) for all *x*_1_^*n*^ ∈ *H*. If such a homomorphism is surjective, injective, or bijective, it is called an* epimorphism*, a* monomorphism,* or an* isomorphism*.

**Lemma 32 .**

Let (*H*, *f*) and (*H*′, *f*′) be two *n*-ary hypergroups with identities *e* and *e*′, respectively, *φ* : *H* → *H*′ a homomorphism, and *ϕ* : *E* → *E*′ a mapping. Then (*φ*, *ϕ*)(*F*(〈*F*, *A*〉_1_^*n*^))⋐*F*′((*φ*, *ϕ*)〈*F*_1_, *A*_1_〉,…, (*φ*, *ϕ*)〈*F*_*n*_, *A*_*n*_〉) for all 〈*F*,*A*〉_1_^*n*^ ∈ *FS*(*H*, *E*) such that ⋂_*i*=1_^*n*^ *A*_*i*_ ≠ *∅* and (*φ*, *ϕ*)(*F*(〈*F*, *A*〉_1_^*n*^)) = *F*′((*φ*, *ϕ*)〈*F*_1_, *A*_1_〉,…, (*φ*, *ϕ*)〈*F*_*n*_, *A*_*n*_〉) if *ϕ* is injective.

**Proof**

Let 〈*F*,*A*〉_1_^*n*^ ∈ *FS*(*H*, *E*), *ε*′ ∈ *ϕ*(⋂_*i*=1_^*n*^ *A*_*i*_)⊆⋂_*i*=1_^*n*^ *ϕ*(*A*_*i*_), and *x*′ ∈ *H*′. If *φ* ^−1^(*x*′) = *∅*, then *F*′(*φ*(*F*_1_),…, *φ*(*F*_*n*_))(*ε*′)(*x*′) = 0 = *φ*(*F*(*F*_1_^*n*^)(*ε*′))(*x*′). Otherwise, we have

(46) F′(φ(F1),…,φ(Fn))(ε′)(x′)  =F′(φ(F1)(ε′),…,φ(Fn)(ε′))(x′)  =sup⁡x′∈f′(x1′,…,xn′)min⁡{φ(F1)(ε′)(x1′),…,φ(Fn)(ε′)(xn′)}  =sup⁡x1′,…,xn′∈Im⁡(φ),x′∈f′(x1′,…,xn′)min⁡{sup⁡ε1∈ϕ−1(ε′)∩A1,φ(x1)=x1′F1(ε1)(x1),…,sup⁡εn∈ϕ−1(ε′)∩An,φ(xn)=xn′Fn(εn)(xn)}  =sup⁡x1′,…,xn′∈Im⁡(φ),x′∈f′(x1′,…,xn′),ε1∈ϕ−1(ε′)∩A1,…,εn∈ϕ−1(ε′)∩Anmin⁡{F1(ε1)(x1),…,Fn(εn)(xn)}  ≥sup⁡x′∈f′(φ(x1),…,φ(xn)),ε∈ϕ−1(ε′)∩∩i=1nAimin⁡{F1(ε)(x1),…,Fn(ε)(xn)}  =sup⁡x′∈φ(f(x1,…,xn)),ε∈ϕ−1(ε′)∩∩i=1nAimin⁡{F1(ε)(x1),…,Fn(ε)(xn)}  =sup⁡x∈φ−1(x′),x∈f(x1,…,xn),ε∈ϕ−1(ε′)∩∩i=1nAimin⁡{F1(ε)(x1),…,Fn(ε)(xn)}  =sup⁡x∈φ−1(x′),ε∈ϕ−1(ε′)∩∩i=1nAiF(F1n(ε))(x)=sup⁡x∈φ−1(x′),ε∈ϕ−1(ε′)∩∩i=1nAiF(F1n)(ε)(x)=φ(F(F1n)(ε′))(x′).

Hence (*φ*, *ϕ*)(*F*(〈*F*, *A*〉_1_^*n*^))⋐*F*′((*φ*, *ϕ*)〈*F*_1_, *A*_1_〉,…, (*φ*, *ϕ*)〈*F*_*n*_, *A*_*n*_〉). If *ϕ* is injective, then the symbol “≥” in the above proof can be replaced with “=”; hence (*φ*, *ϕ*)(*F*(〈*F*, *A*〉_1_^*n*^)) = *F*′((*φ*, *ϕ*)〈*F*_1_, *A*_1_〉,…, (*φ*, *ϕ*)〈*F*_*n*_, *A*_*n*_〉).

**Theorem 33 .**

Let (*H*, *f*) and (*H*′, *f*′) be two commutative *n*-ary hypergroups with identities *e* and *e*′, respectively, *φ* : *H* → *H*′ a homomorphism, and *ϕ* : *E* → *E*′ an injective mapping. Let 〈*F*, *A*〉 be an (∈_*γ*_, ∈_*γ*_∨*q*_*δ*_)-fuzzy *n*-ary soft subhypergroup over *H* which has the sup-property. Then, (*φ*, *ϕ*)〈*F*, *A*〉 is an (∈_*γ*_, ∈_*γ*_∨*q*_*δ*_)-fuzzy *n*-ary subhypergroup over *H*′. If *φ* is an epimorphism from *H* onto *H*′ and 〈*F*, *A*〉 is invertible, then (*φ*, *ϕ*)〈*F*, *A*〉 is also invertible.

**Proof**

Assume that 〈*F*, *A*〉 is an (∈_*γ*_, ∈_*γ*_∨*q*_*δ*_)-fuzzy *n*-ary soft subhypergroup over *H* which has the sup-property and *ε*′ ∈ *ϕ*(*A*). Then there exists a unique *ε* ∈ *A* such that *ϕ*(*ε*) = *ε*′ and we have the following.

(1) It is clear that max⁡{*φ*(*F*)(*ε*′)(*e*′), *γ*} ≥ min⁡{*φ*(*F*)(*ε*′)(*x*′), *δ*} for all *x*′ ∈ *H*.
(2) By Lemma 32, we have
  (47) F′(φ(F)(ε′),…,φ(F)(ε′)︸n) =φ(F(F,…,F︸n))(ε′)⊆∨q(γ,δ)φ(F)(ε′).
(3) Let *y*′, *y*_1_′,…, *y*_*n*−1_′ ∈ *H*′. If *φ* ^−1^(*y*_*j*_′) = *∅*  (1 ≤ *j* ≤ *n* − 1) or *φ* ^−1^(*y*′) = *∅*, then
  (48) max⁡{φ(F)(ε′)(x′),γ}≥0 =min⁡{φ(F)(ε′)y1′yn−1′,φ(F)(ε′)(y′),δ}

for *y*′ ∈ *f*′(*x*′, *y*_1_′,…, *y*_*n*−1_′). Otherwise, there exist *y*, *y*_1_^*n*−1^ ∈ *H* such that *φ*(*y*) = *y*′, *φ*(*y*_*i*_) = *y*_*i*_′, *φ*(*F*)(*ε*′)(*y*′) = *F*(*ε*)(*y*), and *φ*(*F*)(*ε*′)(*y*_*i*_′) = *F*(*ε*)(*y*_*i*_) for all 1 ≤ *i* ≤ *n* − 1 since 〈*F*, *A*〉 has the sup-property. Now, for *y*, *y*_1_^*n*−1^ ∈ *H*, since 〈*F*, *A*〉 is an (∈_*γ*_, ∈_*γ*_∨*q*_*δ*_)-fuzzy soft *n*-ary subhypergroup over *H*, there exists *x* ∈ *H* such that *y* ∈ *f*(*x*, *y*_1_^*n*−1^) and max⁡{*F*(*ε*)(*x*), *γ*} ≥ min⁡{*F*(*ε*)_*y*_1__^*y*_*n*−1_^, *F*(*ε*)(*y*), *δ*}. Hence we have

(49) y′=φ(y)∈φ(f(x,y1n−1))=f′(φ(x),φ(y1),…,φ(yn−1))=f′(φ(x),y1′,…,yn−1′),max⁡{φ(F)(ε′)(φ(x)),γ} =max⁡{sup⁡φ(a)=φ(x)F(ε)(a),γ}≥max⁡{F(ε)(x),γ} ≥min⁡{F(ε)y1yn−1,F(ε)(y),δ} =min⁡{φ(F)(ε′)y1′yn−1′,φ(F)(ε′)(y′),δ}.

Summing up the above analysis, (*φ*, *ϕ*)(〈*F*, *A*〉) is an (∈_*γ*_, ∈_*γ*_∨*q*_*δ*_)-fuzzy soft *n*-ary subhypergroup over *H*′.

Next assume that *φ* is an epimorphism from *H* onto *H*′ and that 〈*F*, *A*〉 is an invertible (∈_*γ*_, ∈_*γ*_∨*q*_*δ*_)-fuzzy soft *n*-ary subhypergroup over *H*. Then, for any *x*′, *y*′ ∈ *H*′, we have

(50) max⁡{min⁡{F′(x′,φ(F)(ε′),…,φ(F)(ε′)︸n−1)(y′),δ},γ} =max⁡{min⁡{sup⁡φ(x)=x′F′(φ(x),φ(F)(ε′),…,φ(F)(ε′)︸n−1)(y′),δ},γ} =max⁡{min⁡{sup⁡φ(x)=x′φ(F(1x~,F,…,F︸n−1))(ε′)(y′),δ},γ} =max⁡{min⁡{sup⁡φ(x)=x′,φ(y)=y′F(x,F(ε),…,F(ε)︸n−1)(y),δ},γ} =sup⁡φ(x)=x′,φ(y)=y′max⁡{min⁡{F(x,F(ε),…,F(ε)︸n−1)(y),δ},γ}.

Similarly, we have

(51) max⁡{min⁡{F′(y′,φ(F)(ε′),…,φ(F)(ε′)︸n−1)(x′),δ},γ}  =sup⁡φ(x)=x′,φ(y)=y′max⁡{min⁡{F(y,F(ε),…,F(ε)︸n−1)(x),δ},γ}.

It follows that

(52) max⁡{min⁡{F′(x′,φ(F)(ε′),…,φ(F)(ε′)︸n−1)(y′),δ},γ}  =max⁡{min⁡{F′(y′,φ(F)(ε′),…,φ(F)(ε′)︸n−1)(x′),δ},γ}

since $\max\left\{\min\left\{F\left(x,\underset{n-1}{\underset{︸}{F\left(\varepsilon \right),\ldots ,F\left(\varepsilon \right)}}\right)\left(y\right),\delta \right\},\gamma \right\}=\max\left\{\min\left\{F\left(y,\underset{n-1}{\underset{︸}{F\left(\varepsilon \right),\ldots ,F\left(\varepsilon \right)}}\right)\left(x\right),\delta \right\},\gamma \right\}$.

Therefore, (*φ*, *ϕ*)〈*F*, *A*〉 is invertible.

**Theorem 34 .**

Let (*H*, *f*) and (*H*′, *f*′) be two commutative *n*-ary hypergroups with identities *e* and *e*′, respectively, *φ* : *H* → *H*′ a homomorphism, and  *ϕ* : *E* → *E*′ a mapping. Let 〈*F*′, *A*′〉 be an (∈_*γ*_, ∈_*γ*_∨*q*_*δ*_)-fuzzy soft *n*-ary subhypergroup over *H*′. Then (*φ*,*ϕ*)^−1^〈*F*′, *A*′〉 is an (∈_*γ*_, ∈_*γ*_∨*q*_*δ*_)-fuzzy soft *n*-ary subhypergroup over *H*. If both *φ* and *ϕ* are injective and 〈*F*′, *A*′〉 is an (∈_*γ*_, ∈_*γ*_∨*q*_*δ*_)-fuzzy soft (invertible) *n*-ary subhypergroup over *H*′, then (*φ*,*ϕ*)^−1^〈*F*′, *A*′〉 is an (∈_*γ*_, ∈_*γ*_∨*q*_*δ*_)-fuzzy soft (invertible) *n*-ary subhypergroup over *H*.

**Proof**

Assume that 〈*F*′, *A*′〉 is an (∈_*γ*_, ∈_*γ*_∨*q*_*δ*_)-fuzzy soft *n*-ary subhypergroup over *H*′. Let *ε* ∈ *ϕ* ^−1^(*A*′). Then we have the following.

(1) For any *x* ∈ *H*, max⁡{*φ* ^−1^(*F*′)(*ε*)(*e*), *γ*} = max⁡{*F*′(*ϕ*(*ε*))(*φ*(*e*)), *γ*} ≥ min⁡{*F*′(*ϕ*(*ε*))(*φ*(*x*)), *δ*} = min⁡{*φ* ^−1^(*F*′)(*ε*)(*x*), *δ*}.
(2) Let *x*, *x*_1_^*n*^ ∈ *H* be such that *x* ∈ *f*(*x*_1_^*n*^). Then *φ*(*x*) ∈ *φ*(*f*(*x*_1_^*n*^)) = *f*′(*φ*(*x*)_1_^*n*^) and so
  (53) max⁡{φ−1(F′)(ε)(x),γ}=max⁡{F′(ϕ(ε))(φ(x)),γ}≥min⁡{F′(ϕ(ε))(φ(x1)),…,F′(ϕ(ε))(φ(xn)),δ}=min⁡{φ−1(F′)(ε)x1xn,δ}.

It follows that max⁡{inf⁡_*x*∈*f*(*x*_1_^*n*^)_⁡*φ* ^−1^(*F*′)(*ε*)(*z*), *γ*} ≥ min⁡{*φ* ^−1^(*F*′)(*ε*)_*x*_1__^*x*_*n*_^, *δ*}.

(3) Let *x*, *y*, *y*_1_^*n*−1^ ∈ *H* be such that *y* ∈ *f*(*x*, *y*_1_^*n*−1^). Then *φ*(*y*) ∈ *f*′(*φ*(*x*), *φ*(*y*_1_),…, *φ*(*y*_*n*−1_)). Since 〈*F*′, *A*′〉 is invertible, by Lemma 25(2), we have
  (54) max⁡{φ−1(F′)(ε)(x),γ} =max⁡{F′(ϕ(ε))(φ(x)),γ} ≥min⁡{F′(ϕ(ε))(φ(y1)),…,F′(ϕ(ε))(φ(yn−1)),    F′(ϕ(ε))(φ(y)),δ} =min⁡{φ−1(F′)(ε)y1yn−1,φ−1(F′)(ε)(y),δ}.

Summing up the above analysis, (*φ*,*ϕ*)^−1^〈*F*′, *A*′〉 is an (∈_*γ*_, ∈_*γ*_∨*q*_*δ*_)-fuzzy *n*-ary soft subhypergroup over *H*. If both *φ* and *ϕ* are injective and 〈*F*′, *A*′〉 is an (∈_*γ*_, ∈_*γ*_∨*q*_*δ*_)-fuzzy soft invertible *n*-ary subhypergroup over *H*′, it is easy to check that (*φ*,*ϕ*)^−1^〈*F*′, *A*′〉 is an (∈_*γ*_, ∈_*γ*_∨*q*_*δ*_)-fuzzy soft invertible *n*-ary subhypergroup over *H*.

## 6. Conclusions

In this paper, our aim is to promote the research and development of fuzzy soft technology by studying fuzzy soft *n*-ary hypergroups which generalize some algebra structures: soft groups, fuzzy *n*-ary hypergroups, fuzzy hypergroups, and so on. The goal is to explain new methodological development in *n*-ary hypergroups which will also be of growing importance in the future. The results presented in this paper can hopefully provide more insight into and a full understanding of fuzzy soft set theory and algebraic hyperstructures. Our future work on this topic will focus on studying some other classes of fuzzy soft *n*-ary hypergroups.
